# Low-Jitter Clock Receivers for Fast Timing Applications

**DOI:** 10.3390/s25072284

**Published:** 2025-04-03

**Authors:** Carl Grace, Maurice Garcia-Sciveres, Timon Heim, Amanda Krieger

**Affiliations:** Lawrence Berkeley National Laboratory, Berkeley, CA 94720, USA; mgarcia-sciveres@lbl.gov (M.G.-S.); theim@lbl.gov (T.H.); akrieger@lbl.gov (A.K.)

**Keywords:** time-to-digital convertor, high-energy physics, instrumentation, analog integrated circuits, low-jitter clocking, medical imaging, positron emission tomography

## Abstract

Precision timing is a key requirement for emerging 4D particle tracking, Positron Emission Tomography (PET), beam and fusion plasma diagnostics, and other systems. Time-to-Digital Converters (TDCs) are commonly used to provide digital estimates of the relative timing between events, but the jitter performance of a TDC can be no better than the performance of the circuits that acquire the pulses and deliver them to the TDC. Several clock receiver and distribution circuits were evaluated, and a differential amplifier with resistive loads driving a pseudo-differential clock distribution network, developed using design guidelines for radiation tolerance and cryogenic compatibility, was fabricated as part of three prototypes: an analog front-end testbed chip for high-precision timing pixel readout, a dedicated TDC evaluation chip, and a Low-Gain Avalanche Detector (LGAD) readout circuit. Based on TDC measurements of the prototypes, we infer that the jitter added by the clock receiver and distribution circuits is less than 2.25 ps-rms. This performance meets the requirements of many future precision timing systems. The clock receiver and on-chip pseudo-differential driver were fabricated in commercial 28-nm CMOS technology and occupy 2288 µm^2^.

## 1. Introduction

A number of emerging sensor and detector modalities require precision timing systems in order to realize their potential. As an example, future high-energy particle colliders set extreme and unique challenges for their detectors in order to reach higher physics discovery potential or unprecedented measurement precision. A development common to all potential future collider experiments is the need to evolve current tracking detectors delivering 3-dimensional spatial information to 4-dimensional tracking detectors by the addition of high-precision timing information.

The Energy Frontier in High-Energy Physics (HEP) will be based on high-intensity and still higher-energy proton colliders for decades to come [1]. Adding 4D particle tracking will be a critical component of a tracking system able to reliably reconstruct collisions in such future accelerators—it will be a crucial enabling technology for future colliders, such as the Future Circular Collider (FCC).

In the proposed FCC-hh future hadron collider, the number of collisions during a single bunch crossing is expected to be five times higher than the predictions for the HL-LHC. In order to perform vertex reconstruction in such an environment, high-precision timing on the order of 10 ps-rms will be required [2]. Similar arguments indicate fast timing is required for other proposed colliders, such as the future muon collider [3] and the future Higgs factory (e.g., FCC-ee or ILC) [4].

Fast precision timing is also of interest to next-generation Positron Emission Tomography (PET) systems, particularly whole-body systems, where improved timing will enable more compact, lower-cost, and higher-performance systems [5]. The improved timing is required to take advantage of the capabilities of new Silicon Photomultipliers (SiPMs) [6] that enable new Time-of-Flight (TOF) approaches to measuring gamma rays. In TOF imaging, the source of the gamma rays can be localized by comparing timestamps from SiPM imagers on opposite sides of the patient [7]. In TOFPET imaging, the spatial resolution directly depends on the timing resolution [8], driving interest in precision timing.

New sensor types with unprecedentedly fast rise times are emerging that will enable new science. The Low-Gain Avalanche Diode (LGAD) [9], for instance, has a very fast detection time and can exhibit rise times in the tens of ns [10]. LGADs are finding use in High-Energy Physics applications, such as in the Endcap Timing Layer in the Compact Muon Solenoid (CMS) experiment [11], due to their high speed and radiation tolerance.

Emerging demands in fusion diagnostics are also driving the development of fast diamond sensors that can simultaneously provide extremely fast rise times, wide dynamic range, and suitable radiation tolerance for the in situ imaging of particle beams [12] and plasma burns in inertial confinement fusion [13].

A key circuit to enable the precision timing in all of these applications is the Time-to-Digital Converter (TDC), which is widely used in collider experiments. Leading-edge TDCs can provide jitter performance better than 10 ps-rms and require multi-GHz clocks [14]. In the early design phase of a pixel readout chip, it is advantageous to reduce the complexity of prototype chips to focus on enhancing specific components of it; therefore, no phased-locked loop circuit was included in the implemented prototypes. The timing performance of a TDC can be no better than the jitter performance of the clock that is used by the TDC. Therefore, the jitter performance of the clock receiver and clock distribution network that define the TDC time base are critical when specifying a precision timing system. The jitter performance is quantified using a Figure-of-Merit (FOM) adapted from [15].

This work describes the exploration of a number of clock receiver architectures to develop a clock receiver and distribution network to drive a precision TDC with low jitter.

## 2. Materials and Methods

### 2.1. Technology Selection

There is considerable interest in using 28 nm CMOS technology for the next generation of collider and accelerator experiments. Preliminary studies indicate that its radiation hardness could be sufficient for its use in the High-Luminosity Large Hadron Collider (HL-LHC) environment [16]. The desire to be compatible with future collider readout ASIC developments drove the process selection.

### 2.2. Receiver Architecture

There are numerous ways that a high-speed differential clock can be received and distributed around a chip. The most straight-forward is to use an industry standard Low-Voltage Differential Signaling (LVDS) receiver and then distribute CMOS logic levels. The various architectures depicted here are also implementing the differential-to-signal-ended conversion function, but this function can be carried out later in the signal chain (for instance, if there is a desire for differential clock distribution internal to the chip).

#### 2.2.1. LVDS

**Figure 1:**
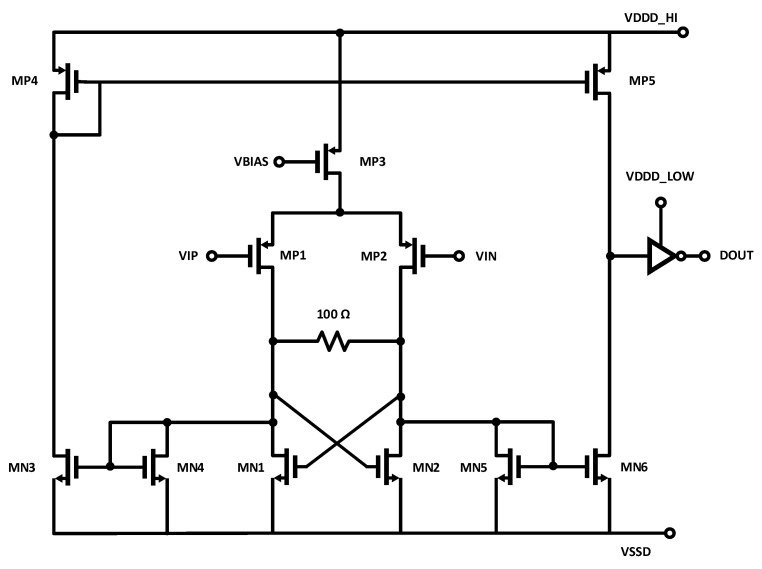
Schematic of typical LVDS receiver circuit.

The schematic of a typical LVDS receiver is shown in Figure 1 [17]. The 3.5 mA nominal LVDS loop current is dropped across a 100-Ω differential sense resistor, which both terminates the communication channel and develops the differential input voltage. This differential input voltage causes the input differential pair to steer the bias current to a cross-coupled load. This cross-coupled load implements positive feedback, which speeds up the switching. The signal is then presented to a digital logic inverter, which converts the signal to CMOS logic levels.

The cross-coupled, positive-feedback load increases the slope at the output of the first stage of the receiver, reducing jitter. However, it also adds noise, which increases jitter. The LVDS circuit is popular in part because it is quite robust and tolerant to errors such as mismatches in termination resistance and loop current. While the positive feedback increases the slope at the output of the first stage, it also significantly increases the bandwidth of the circuit, with the result being increased noise and jitter compared to other receiver techniques.

#### 2.2.2. Differential-Pair with Resistive Load

**Figure 2:**
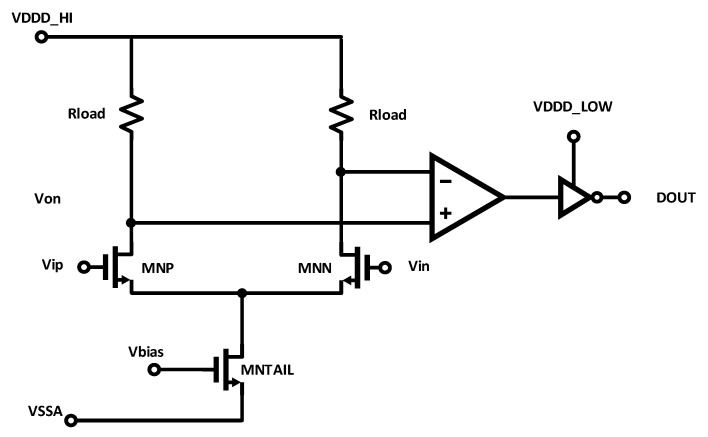
Schematic of DPRL clock receiver circuit.

The schematic of an alternative clock receiver, a differential pair with a resistive load (DPRL), is shown in Figure 2. The key idea here is that a resistive load generates less input-referred noise for a given transconductance than a conventional, actively loaded amplifier [18]. Like the LVDS receiver, after the input differential pair senses the voltage difference, the signal is passed to a comparator and then one or more inverters to generate CMOS logic levels. One disadvantage of this technique is its low gain, which makes the noise of downstream stages more significant to the overall jitter. This solution also typically dissipates relatively high power.

#### 2.2.3. Multi-Stage Amplification

**Figure 3:**
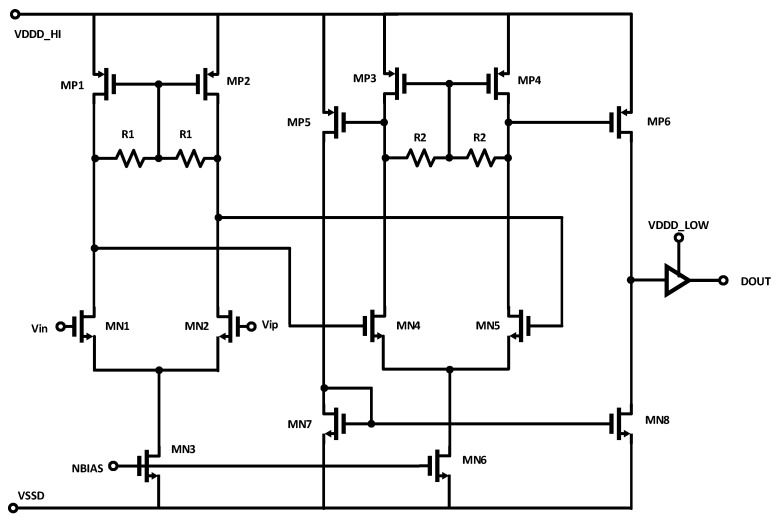
Multi-stage amplification for clock reception.

A third receiver architecture, a multi-stage amplifier with resistive loads, is shown in Figure 3. The goal here is to combine the low noise of the DPRL with the high gain and fast switching of the LVDS receiver. This is achieved by distributing the gain over a series of fast, low-gain stages, with a final conversion to CMOS logic levels. This approach is similar to what is often used in high-speed comparator circuits [19]. The benefit here is that cascades of low-gain stages tend to allow for higher operating frequencies in a given technology, with the trade-off that the noise in increased due to the additional stages.

#### 2.2.4. Clock Distribution

**Figure 4:**
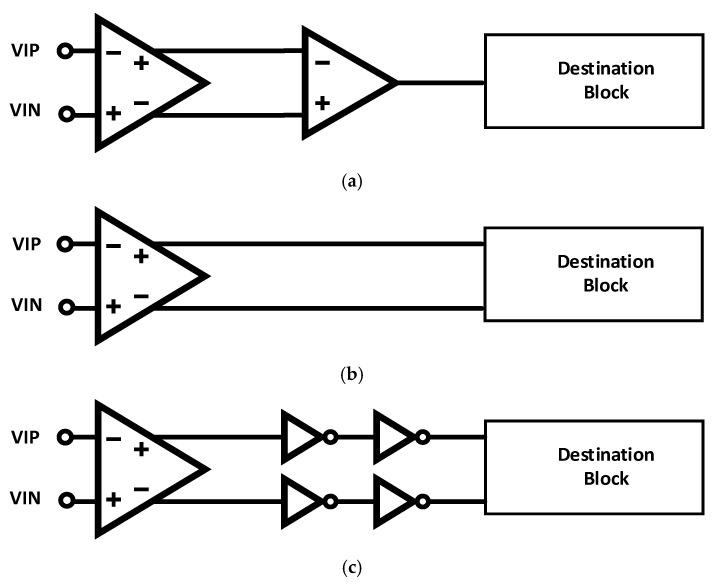
Three methods of clock distribution evaluated: (**a**) fully differential clock reception and singled-ended clock distribution; (**b**) fully differential clock distribution from the receiver all the way to the TDC; and (**c**) pseudo-differential clock distribution.

Regardless of the front-end architecture used, the buffered clock needs to be distributed to the TDC circuits that will use it. Three techniques are evaluated here: single-ended CMOS distribution, Current-Mode Logic (CML) fully differential clock distribution, and pseudo-differential CMOS clock distribution, where opposite CMOS logic levels are transmitted together using two conductors. The three clock distribution architectures are shown in block-diagram form in Figure 4.

#### 2.2.5. Simulated Comparison

All three approaches to a clock receiver front-end circuit were simulated extensively for power dissipation, jitter, and high-frequency performance. Jitter simulation was carried out both in the frequency domain and the time domain, using Periodic Steady State and Transient Noise simulation techniques, respectively.

The results of the simulation study of the three architectures are shown in Table 1.

Based on the simulation study, it was determined that the best performance was given by a combination of the DPRL circuit coupled with a pseudo-differential clock distribution network. While the LVDS receiver had slightly lower power consumption, its jitter was about ten times as large as the jitter simulated for the DPRL circuit. This result agrees with studies indicating that CMOS clock distribution provides better jitter performance in scaled technologies than CML-based clock distribution or resonant clock distribution networks [20].

### 2.3. Prototype

To demonstrate the performance of the clock receiver and distribution circuits, the circuit was included on a prototype detector readout front end developed by Lawrence Berkeley National Laboratory (LBNL) and in two prototype multi-channel TDC integrated circuits developed by the SLAC National Accelerator Laboratory. The LBNL prototype was an analog front-end testbed for high-precision timing pixel readout [21]. One of the SLAC prototypes was dedicated to characterizing the TDC and has been described in [22]; the other prototype was developed for the readout of LGADs [23]. The performance of the clock receiver and distribution circuits as measured in the context of those systems is described here. The prototypes were implemented in commercial 28 nm CMOS technology.

#### 2.3.1. Schematic Design

**Figure 5:**
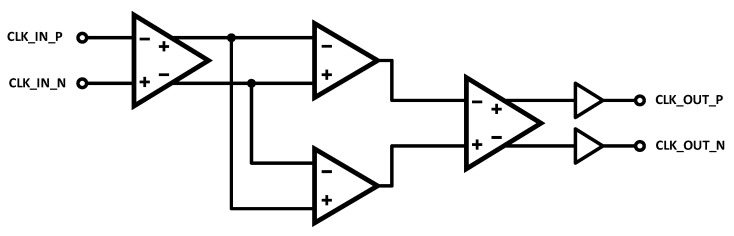
Block diagram of clock receiver and pseudo-differential clock distribution.

A simplified block diagram of the implemented clock receiver and distribution network is shown in Figure 5. A DPRL drives two differential-to-single-ended converters, which are then buffered by a pseudo-differential amplifier. The clock is distributed to the TDCs on the prototype using an H-network [24], and the CMOS buffers in Figure 5 denote pseudo-differential clock repeaters placed throughout the network.

**Figure 6:**
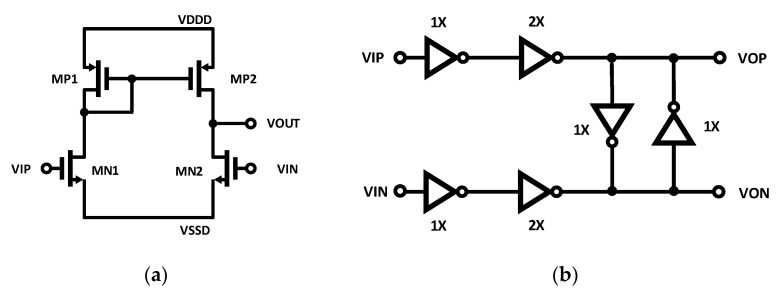
Clock distribution circuits. (**a**) Differential-single-ended converter; (**b**) pseudo-differential CMOS buffer.

The schematic of the differential-to-single-ended converters and the pseudo-differential CMOS buffers is shown in Figure 6a,b, respectively. The fully differential output from the clock receiver is converted to pseudo-differential CMOS levels by the differential-to-single-ended converter. Matched CMOS inverters are used to implement the pseudo-differential CMOS buffers. The cross-coupled inverters serve to mitigate duty cycle distortion [25]. The transistors use larger than minimum lengths in order to enhance the radiation tolerance and cryogenic capability of the clock receiver for future use in radiation fields or cold environment [16]. These design techniques typically trade off functionality and survivability for performance, area, and power dissipation.

#### 2.3.2. Layout Design

**Figure 7:**
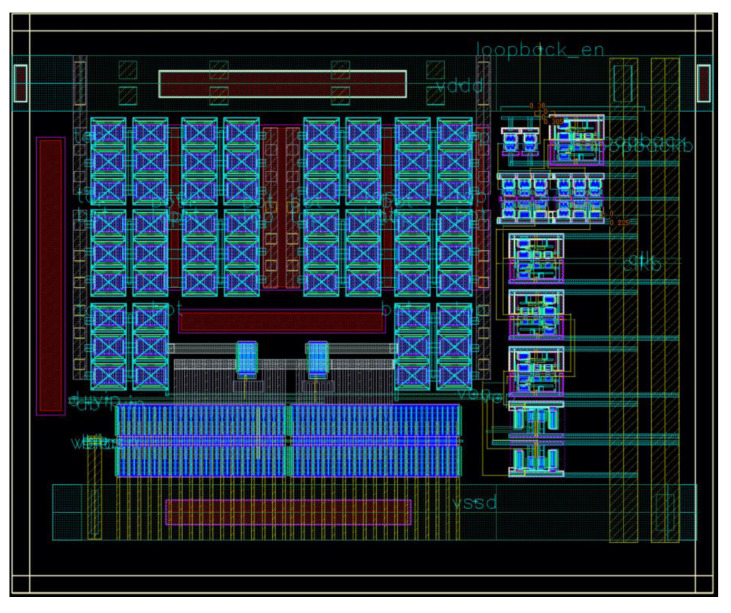
Layout of clock receiver and pseudo-differential amplifier. The area is 52 µm by 44 µm.

The layout of the clock receiver and the pseudo-differential amplifier in the clock distribution network is shown in Figure 7. The area of the macro is approximately 52 µm by 44 µm (2288 µm^2^).

To reduce induced jitter, the clock receiver and the pseudo-differential clock amplifier are placed in deep n-wells to isolate them from dynamic currents and powered by a dedicated I/O power supply. The H-network is laid out to balance the length of the wires to each TDC on the prototype chip, and the lines are shielded to reduce crosstalk and noise pick up.

#### 2.3.3. Transient Jitter Simulation

**Figure 8:**
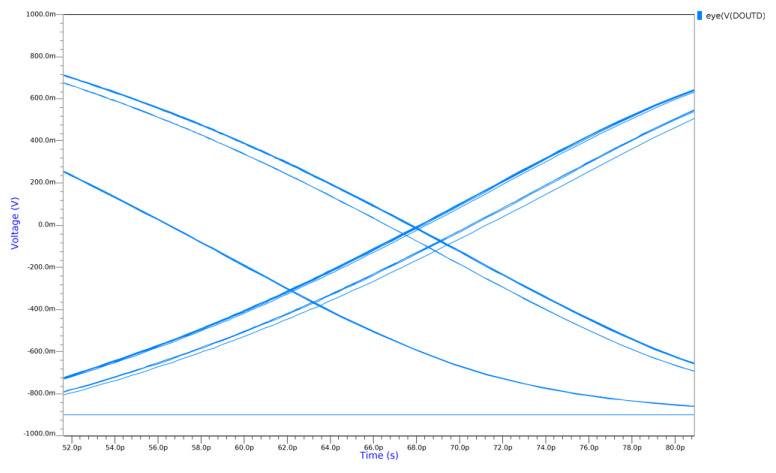
Post-layout transient noise simulation showing a zoom of the crossover point of the eye diagram. The rms jitter in this simulation is approximately 1.2 ps-rms.

The simulated jitter is shown in Figure 8. This post-layout simulation comprised 100 Transient Noise simulations zoomed into the crossover point of the eye diagram. The noise of the clock receivers is included in this simulation, and the input signal was a PRBS7 sequence [26]. The peak-to-peak jitter in this simulation is about 8 ps-rms, which translates to an rms-jitter of approximately 1.2 ps-rms, assuming a Gaussian distribution. The jitter was also simulated using Periodic Steady State simulation with similar results.

#### 2.3.4. Die Photo

**Figure 9:**
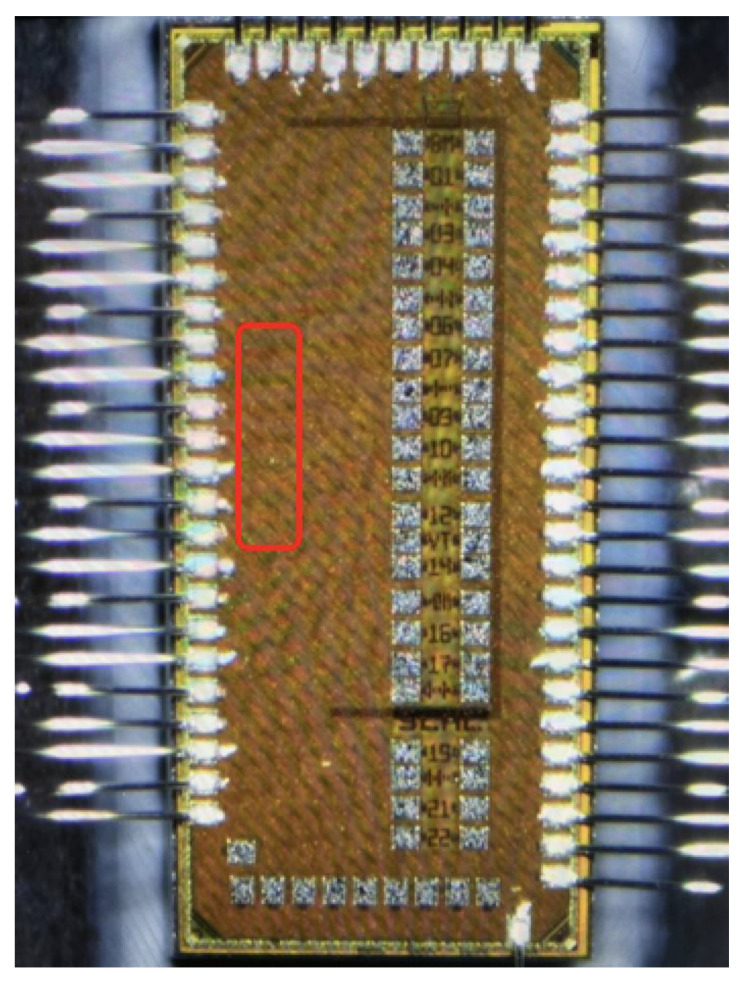
Die Photo of TDC evaluation prototype. Clock receivers are in outlined area.

A die photo of the TDC prototype described in [22] is shown in Figure 9. The location of the clock receiver circuits is outlined in red. The clock is then distributed to arrays of TDCs and other test structures on the right side of the die.

## 3. Results

The multi-channel TDC prototype that included the clock receivers and distribution network was tested and characterized at SLAC National Accelerator Laboratory and reported in [22,23]. The clock receiver was also demonstrated to be functional in [21], but jitter calculations of the clock receiver were not carried out.

### 3.1. Test Setup

**Figure 10:**
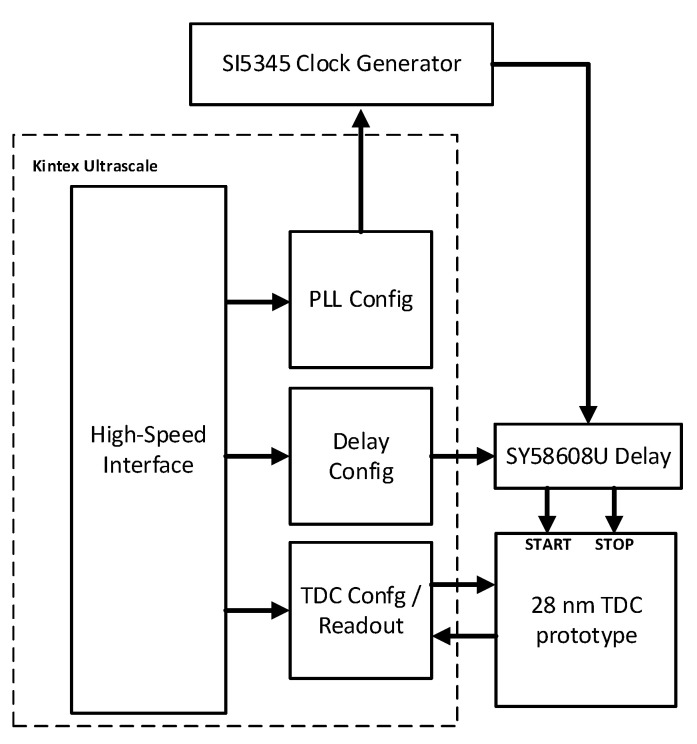
Block diagram of the test setup used to evaluate TDC and clock receiver jitter performance of the clock receiver and TDC prototype.

**Figure 11:**
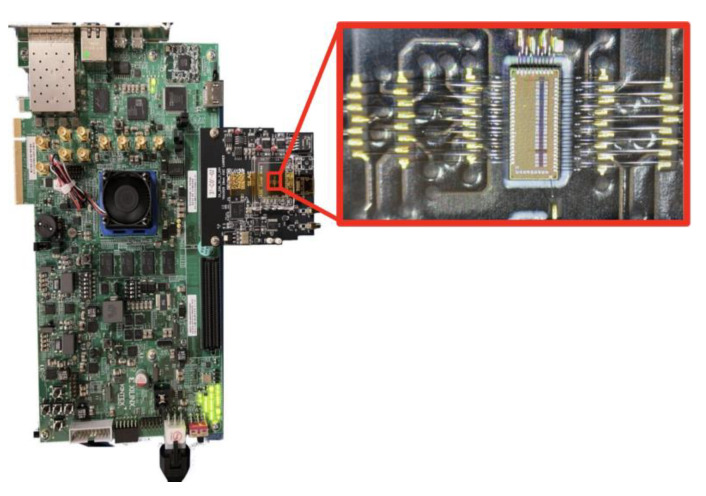
Photograph of test setup used to evaluate TDC and clock receiver jitter. The prototype ASIC is bonded to an FMC and mounted onto a commercial KCU105 development board. Figure is adapted from [22] with permission.

The test setup used to evaluate the TDC and clock receiver circuits is show in Figure 10. The prototype chip is bonded to a custom FMC card and them mounted onto a commercial AMD (Sunnyvale, CA, USA) KCU105 Kintex Ultrascale development board. The clocks were generated using a Skyworks Solutions (Irvine, CA, USA) SI5345 clock generator, and the prototype chip was attached to the development board using a mezzanine card. An external programmable delay with 5 ps resolution and 2.56 ns range was used to generate start and stop pulses for the TDC, which were then buffered by a Microchip Technology (Chandler, AZ, USA) SY58608U LVDS buffer chip. The clock receiver reported here was used to receive these LVDS start and stop pulses and to distribute them to the TDCs on the prototype integrated circuit. A photograph of the test setup is shown in Figure 11.

### 3.2. Measured Results

#### Time-of-Arrival Jitter

The most relevant jitter measurement for TDCs used in fast timing applications is the single-shot Time-of-Arrival (TOA) measurement. In this measurement, fast start and stop pulses were generated, and the stop pulses were delayed by a programmable, commercial delay.

The measured jitter in this test system has three components: the jitter of the pulse generator and delay line, the jitter of the clock receiver, and the jitter of the TDC itself. While it is impossible to fully isolate the jitter of the clock receiver from the total jitter measured, it is possible to put upper bounds on the clock receiver jitter.

The jitter of the test stimulus system (comprising a fast pulse generator and a commercial programmable delay) was measured using an oscilloscope to be approximately 6 ps-rms. The measurement uncertainty in this system was approximately 500 fs-rms. This measurement uncertainty includes the jitter between the original start and stop pulses, which is less than 100 fs-rms (based on the SI5345 data sheet [22]). Therefore, the uncertainty is dominated by the commercial delay line.

**Figure 12:**
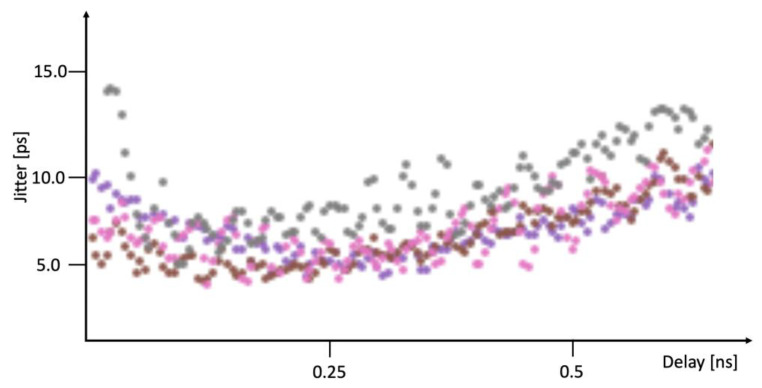
Measured Time-of-Arrival (TOA) jitter as a function of delay. Plot generated using data from [22] with permission.

The measurement of the overall TOA jitter is shown in Figure 12, based on data from [22]. The jitter of the full test system (stimulus, clock receiver, and TDC) was measured to be approximately 8 ps-rms when delay values from 0 to 0.5 ns are considered. Delays higher than about 0.5 ns suffered increased jitter due to inadequate power supply filtering on the internal digital-to-analog converters (DACs) that controlled the programmable digital delay.

Because of the jitter in the test setup, the clock receiver, and the TDC are mutually uncorrelated; they can be subtracted in quadrature, and the resultant jitter in the clock receiver and TDC combination is approximately 5.3 ps-rms. Increasing the power supply of the clock receiver and increasing its bias current did not have a measurable effect on overall jitter. Therefore, the maximum jitter that could be attributed to the clock receiver would be similar in magnitude to the uncertainty of the measurement. To add 500 fs-rms to the overall jitter of the clock receiver and TDC combination, then, the upper bound on the clock receiver jitter is approximately 2.25 ps-rms. It is likely it is significantly smaller than this (in fact it was simulated to be 1.2 fs-rms, as shown in Figure 8, although the clock distribution network also contributes jitter), but this measurement gives an upper bound.

Based on the TDC measurements presented in [22,23], we can infer that the jitter performance of the clock receiver circuit is better than about 2.25 ps-rms. This is a worst-case estimate of the clock receiver jitter (as it assumes none of the measured jitter is due to interference or noise) and is limited by the capabilities of the test equipment used to measure the circuits. These measurements are also consistent with simulation.

### 3.3. Comparison with Other Work

A comparison of both simulated and measured jitter performance is shown in Table 2. The performance is compared using a FOM modified from the FOM proposed in [15]. The FOM is designed to evaluate the jitter performance of Phase-Locked Loops (PLLs) in the context of power dissipation and operating speed. This FOM can also be used to compare low-jitter clock receivers. The figure of merit is defined as:

$$FOM=10log\left[{\left(\frac{\sigma_{t}}{1\;ps}\right)}^{2}\cdot \frac{P}{1\;mW}\cdot \frac{1\;GHz}{f_{clk}}\right]$$

where $\sigma_{t}$ is the rms jitter in ps, *P* is the power dissipation in mW, and ${\;f}_{clk}\;$ is the clock frequency at which measurements were taken. The normalization to ps in the rms jitter has been added to reduce the magnitude of the FOM, and frequency has been added (normalized to GHz). For this FOM, smaller is better. The FOM here is normalized to ps here to make it easier to compare across references.

The results indicate that competitive jitter performance can be achieved with the DPRL-based clock receiver even when conservatively designed following design principles for radiation tolerance and cryogenic compatibility. These design principles (for example, larger well definition and device separation, increased minimum length, and reduced supply voltages) tend to compromise the performance of circuits designed for operation in extreme environments. Therefore, this work demonstrates that competitive performance can be obtained even when the clock receivers are designed expressly for cryogenic compatibility and radiation tolerance.

## 4. Conclusions

Measurements indicate that the jitter of the clock receiver is better than 2.25 ps-rms. This jitter performance is consistent with the requirements of future readout integrated circuits for 4D trackers and PET systems.

## Figures and Tables

**Table 1:** Comparison of simulated performance of clock receivers.

Circuit	Frequency [GHz]	rms Jitter [ps]	Power [mW]
LVDS	5.0	12.2	1.1
Multi-stage	5.0	4.9	2.1
DPRL	5.0	1.2	1.8

**Table 2:** Comparison of jitter performance with prior art.

References	Process	Frequency [GHz]	rms Jitter [ps]	Power [mW]	FOM [dB]
[27]	28	3.5	0.1	10	−15.4
[28]	55	3.0	2.14	2.1	5.06
[29]	28	4.0	1.82	1.9	1.97
[30]	65	0.2	0.2	1.9	−4.20
[31]	130	11.2	58.9	1.6	2.69
This Work	28	5.0	2.25 ^1^	1.8	2.61

^1^ Maximum jitter of clock receiver, inferred from TDC measurement.

## Data Availability

No new data were created or analyzed in this study. Data sharing is not applicable to this article.

## Abbreviations

The following abbreviations are used in this manuscript:

CMOS	Complementary Metal Oxide Semiconductor
CMS	Compact Muon Solenoid
DAC	Digital-to-Analog Converter
DPRL	Differential Pair with Resistive Load
FCC	Future Circular Collider
FMC	FPGA Mezzanine Card
FPGA	Field Programmable Gate Array
FOM	Figure-of-Merit
HEP	High-Energy Physics
HL-LHC	High-Luminosity Large Hadron Collider
ILC	International Linear Collider
LGAD	Low-Gain Avalanche Detector
LVDS	Low-Voltage Differential Signaling
PET	Positron Emission Tomography
PLL	Phase-Locked Loop
PRBS	Pseudo-Random Bit Sequence
SiPM	Silicon Photomultipliers
TDC	Time-to-Digital Converter
TOA	Time-of-Arrival
TOF	Time-of-Flight
